# Supplementary figures and images for: Improvement of subsoil physicochemical and microbial properties by short-term fallow practices
Source: PeerJ. 2019 Aug 19;7:e7501. doi: 10.7717/peerj.7501 (PMC6705386; doi:10.7717/peerj.7501)

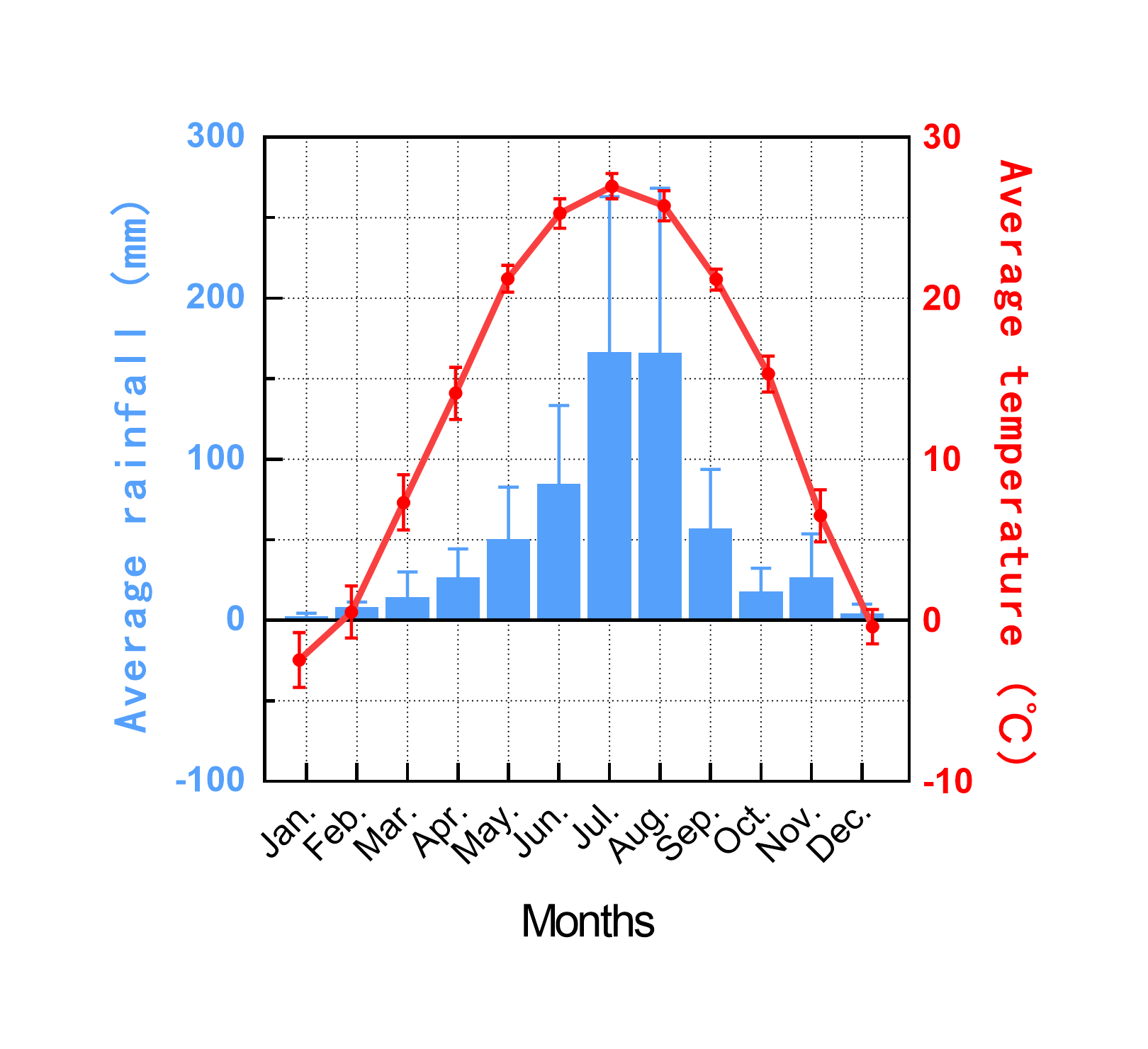

Supplement: Figure S1 [file peerj-07-7501-s001.png]

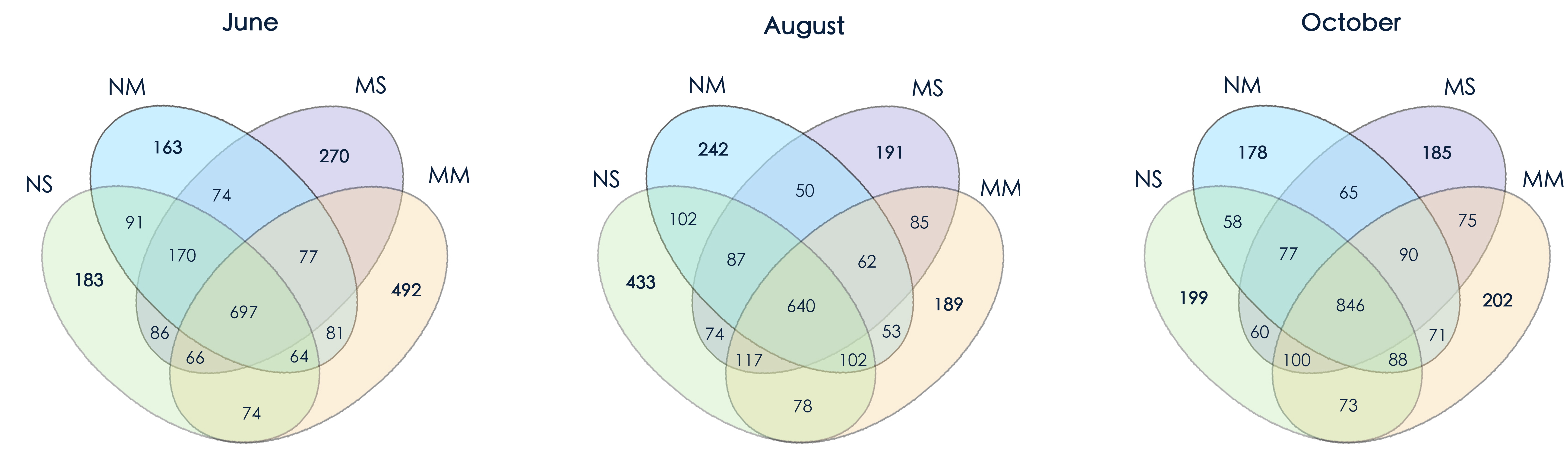

Supplement: Figure S2 — Unique objects in seasons are as follow, spring: 2677, summer:2505, and autumn: 2367. [file peerj-07-7501-s002.png]

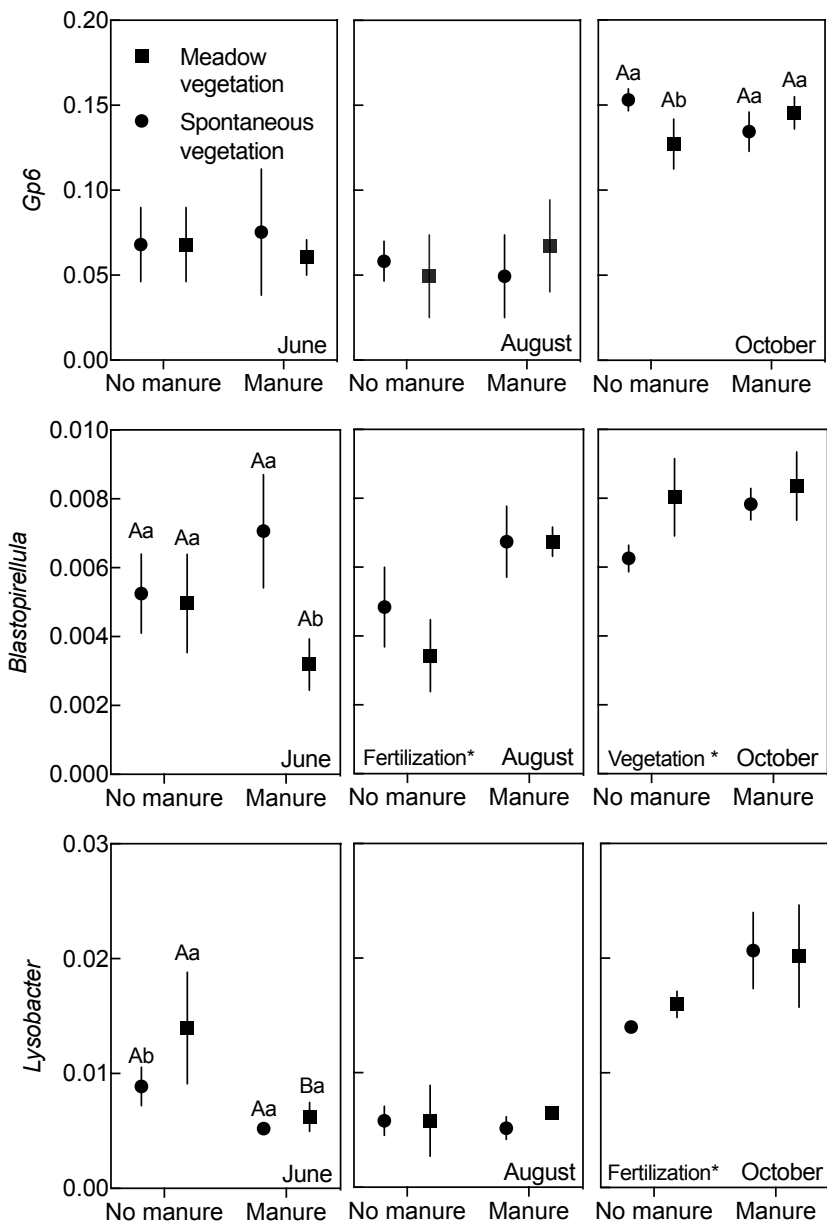

Supplement: Figure S3 — The plots, which were marked upper- and lowercase, represented the interaction factor’s impact was significant. And the uppercase means the fertilization impacts and the lowercase means the vegetation impact (the simple effect test was used to analyze the significance of the difference). The plots which were only impacted by a principal factor, the name of the factor was marked in the upper right corner of each plot. *, ** and *** are used to show statistical significance at the 0.05, 0.01, and 0.001 level, respectively. [file peerj-07-7501-s003.pdf]

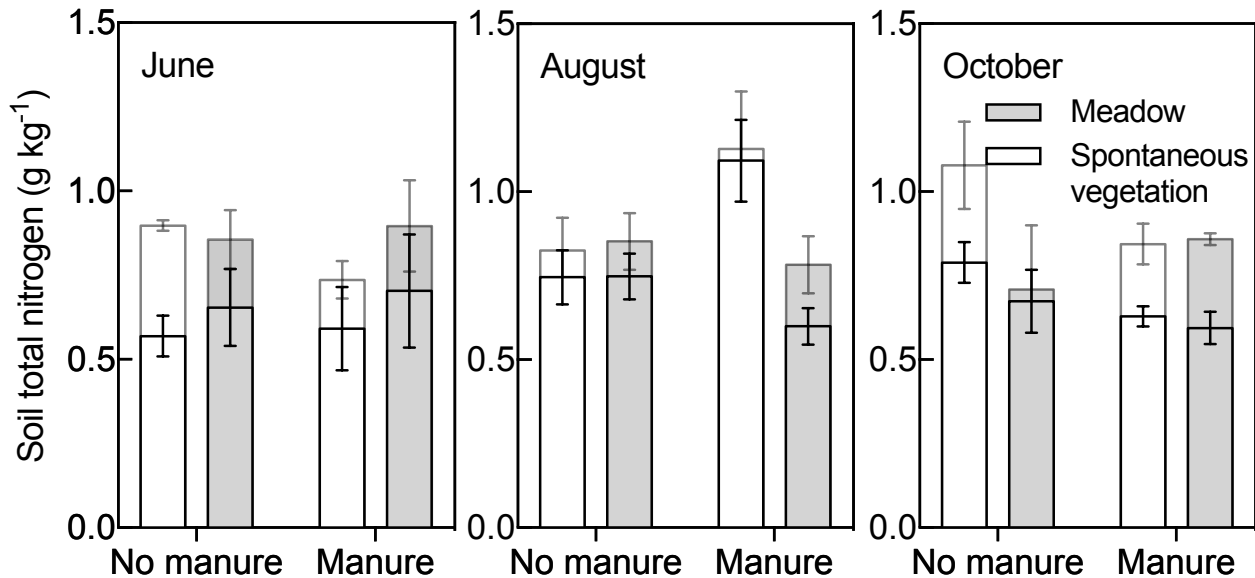

Supplement: Figure S4 — The gray framed column represents the topsoil. [file peerj-07-7501-s004.pdf]
