# Supplementary material for: Improvement of subsoil physicochemical and microbial properties by short-term fallow practices
Source: PeerJ. 2019 Aug 19;7:e7501. doi: 10.7717/peerj.7501 (PMC6705386; doi:10.7717/peerj.7501)
Supplement: Table S1 [file peerj-07-7501-s005.docx]

| Soil variables | Sampling date | Fallowing treatments | Fallowing × Sampling date |
| --- | --- | --- | --- |
| Soil physiochemical properties | |  |  |
| pH | 22.60******* | 1.05 | 1.94 |
| SOC | 11.63******* | 0.67 | 0.65 |
| TN | 34.02******* | 2.45 | 9.20******* |
| C/N | 3.74***** | 1.79 | 5.78****** |
| MBC | 398.22******* | 2.20 | 4.54****** |
| MBN | 312.43******* | 7.99****** | 7.18****** |
| MBC/MBN | 52.97******* | 3.55 | 4.95****** |
| NH_4_^+^-N | 50.97******* | 1.15 | 1.87 |
| Alpha diversity |  |  |  |
| Chao1 Index | 1.28 | 0.96 | 1.35 |
| Coverage | 4.01 | 0.87 | 1.64 |
| Richness | 0.25 | 1.02 | 1.26 |
| Shannon Index | 1.47 | 1.49 | 0.82 |
| Simpson Index | 3.72 | 1.72 | 0.46 |
| Selected genera |  |  |  |
| *Nitrososphaera* | 46.22******* | 2.82 | 3.83***** |
| *Gp10* | 44.75******* | 0.50 | 2.67 |
| *Gp21* | 3.46 | 0.35 | 1.18 |
| *Gp3* | 3.25 | 2.50 | 0.99 |
| *Gp4* | 34.14******* | 1.13 | 0.19 |
| *Gp6* | 60.09******* | 0.86 | 0.55 |
| *Gp7* | 9.60****** | 3.60 | 3.44***** |
| *Gp9* | 5.83***** | 1.30 | 0.97 |
| *Gaiella* | 2.10 | 6.94***** | 3.63***** |
| *Ohtaekwangia* | 24.71******* | 0.003 | 1.60 |
| *Sphaerobacter* | 71.91******* | 3.89 | 3.26***** |
| *Gemmatimonas* | 19.27******* | 1.27 | 0.30 |
| *Nitrospira* | 5.67***** | 3.58 | 3.51***** |
| *Blastopirellula* | 16.62******* | 11.20****** | 4.42****** |
| *Pirellula* | 13.42******* | 1.57 | 1.12 |
| *Geminicoccus* | 39.19******* | 4.44***** | 12.11******* |
| *Ramlibacter* | 14.79****** | 5.68***** | 2.157 |
| *Steroidobacter* | 3.04***** | 1.59 | 0.78 |
| *Luteimonas* | 93.95******* | 0.79 | 2.60 |
| *Lysobacter* | 72.27******* | 1.65 | 5.40****** |
| † *****, ****** and ******* are used to show statistical significance at the 0.05, 0.01, and 0.001 level, respectively. | | | |
