# Supplementary material for: Improvement of subsoil physicochemical and microbial properties by short-term fallow practices
Source: PeerJ. 2019 Aug 19;7:e7501. doi: 10.7717/peerj.7501 (PMC6705386; doi:10.7717/peerj.7501)
Supplement: Table S2 [file peerj-07-7501-s006.docx]

| Soil variables |  | Fertilization | | |  | Vegetation | | |  | Fertilization × Vegetation | | |
| --- | --- | --- | --- | --- | --- | --- | --- | --- | --- | --- | --- | --- |
|  |  | F | df | P |  | F | df | P |  | F | df | P |
| June |  |  |  |  |  |  |  |  |  |  |  |  |
| pH |  | 0.158 | 1 | 0.701 |  | 1.131 | 1 | 0.319 |  | 2.054 | 1 | 0.190 |
| SOC |  | 2.975 | 1 | 0.123 |  | 1.005 | 1 | 0.346 |  | 1.544 | 1 | 0.249 |
| TN |  | 0.256 | 1 | 0.626 |  | 1.938 | 1 | 0.201 |  | 0.036 | 1 | 0.854 |
| C/N |  | 2.983 | 1 | 0.122 |  | 7.889***** | 1 | 0.023 |  | 3.433 | 1 | 0.101 |
| MBC |  | 3.654 | 1 | 0.092 |  | 0.608 | 1 | 0.458 |  | 0.99 | 1 | 0.349 |
| MBN |  | 0.05 | 1 | 0.828 |  | 0.483 | 1 | 0.507 |  | 1.405 | 1 | 0.270 |
| MBC/MBN |  | 2.411 | 1 | 0.159 |  | 0.013 | 1 | 0.912 |  | 4.15 | 1 | 0.076 |
| NH_4_^+^-N |  | 0.643 | 1 | 0.446 |  | 0.191 | 1 | 0.674 |  | 3.396 | 1 | 0.103 |
| August |  |  |  |  |  |  |  |  |  |  |  |  |
| pH |  | 0.003 | 1 | 0.955 |  | 0.065 | 1 | 0.805 |  | 0.003 | 1 | 0.955 |
| SOC |  | 0.397 | 1 | 0.546 |  | 0.149 | 1 | 0.709 |  | 0.001 | 1 | 0.976 |
| TN |  | 4.076 | 1 | 0.078 |  | 25.013******* | 1 | 0.001 |  | 25.474******* | 1 | 0.001 |
| C/N |  | 1.865 | 1 | 0.209 |  | 6.562***** | 1 | 0.034 |  | 10.464***** | 1 | 0.012 |
| MBC |  | 0.327 | 1 | 0.583 |  | 3.299 | 1 | 0.107 |  | 2.71 | 1 | 0.138 |
| MBN |  | 0.563 | 1 | 0.475 |  | 7.278***** | 1 | 0.027 |  | 8.552***** | 1 | 0.019 |
| MBC/MBN |  | 0.092 | 1 | 0.770 |  | 0.037 | 1 | 0.853 |  | 16.336****** | 1 | 0.004 |
| NH_4_^+^-N |  | 1.577 | 1 | 0.245 |  | 4.289 | 1 | 0.072 |  | 0.006 | 1 | 0.942 |
| October |  |  |  |  |  |  |  |  |  |  |  |  |
| pH |  | 8.367***** | 1 | 0.020 |  | 0.294 | 1 | 0.602 |  | 0.328 | 1 | 0.583 |
| SOC |  | 0.581 | 1 | 0.468 |  | 0.844 | 1 | 0.385 |  | 0.023 | 1 | 0.884 |
| TN |  | 11.024***** | 1 | 0.011 |  | 4.306 | 1 | 0.072 |  | 1.225 | 1 | 0.301 |
| C/N |  | 8.867***** | 1 | 0.018 |  | 0 | 1 | 0.997 |  | 0.033 | 1 | 0.860 |
| MBC |  | 1.704 | 1 | 0.228 |  | 2.288 | 1 | 0.169 |  | 0.569 | 1 | 0.472 |
| MBN |  | 8.681***** | 1 | 0.019 |  | 0.881 | 1 | 0.375 |  | 17.742****** | 1 | 0.003 |
| MBC/MBN |  | 2.795 | 1 | 0.133 |  | 2.366 | 1 | 0.163 |  | 28.149******* | 1 | 0.001 |
| NH_4_^+^-N |  | 0.162 | 1 | 0.698 |  | 8.797***** | 1 | 0.018 |  | 0.594 | 1 | 0.463 |
| † *, ** and *** are used to show statistical significance at the 0.05, 0.01, and 0.001 level, respectively.  SOC means soil organic carbon; TN means total nitrogen; C/N means the ratio of organic carbon to total nitrogen; MBC means microbial biomass carbon; MBN means microbial biomass nitrogen; MBC/MBN means the ratio of microbial biomass carbon to microbial biomass nitrogen; NH4+-N represents ammonia nitrogen. | | | | | | | | | | | | |
