# Supplementary material for: Improvement of subsoil physicochemical and microbial properties by short-term fallow practices
Source: PeerJ. 2019 Aug 19;7:e7501. doi: 10.7717/peerj.7501 (PMC6705386; doi:10.7717/peerj.7501)
Supplement: Table S3 [file peerj-07-7501-s007.docx]

|  | | | |
| --- | --- | --- | --- |
| NMDS properties | June and August | August and October | June and October |
| Stress value | 0.117 | 0.135 | 0.074 |
| Dimensions | 2 | 2 | 2 |
