# Supplementary material for: Improvement of subsoil physicochemical and microbial properties by short-term fallow practices
Source: PeerJ. 2019 Aug 19;7:e7501. doi: 10.7717/peerj.7501 (PMC6705386; doi:10.7717/peerj.7501)
Supplement: Table S4 — The P values less than 0.05 are highlighted in bold. [file peerj-07-7501-s008.docx]

|  | | | |
| --- | --- | --- | --- |
| Managements | R^2^ | F | P |
| June |  |  |  |
| Fertilization | **0.1956** | **2.432** | **0.020** |
| Vegetation | 0.1285 | 1.474 | 0.185 |
| Fertilization × Vegetation | **0.2295** | **2.979** | **0.007** |
| August |  |  |  |
| Fertilizer | 0.1324 | 1.527 | 0.077 |
| Vegetation | 0.0753 | 0.815 | 0.645 |
| Fertilization × Vegetation | 0.1078 | 1.209 | 0.224 |
| October |  |  |  |
| Fertilizer | 0.0967 | 1.071 | 0.249 |
| Vegetation | 0.0757 | 0.819 | 0.951 |
| Fertilization × Vegetation | 0.0926 | 1.021 | 0.35 |
