# Supplementary material for: Improvement of subsoil physicochemical and microbial properties by short-term fallow practices
Source: PeerJ. 2019 Aug 19;7:e7501. doi: 10.7717/peerj.7501 (PMC6705386; doi:10.7717/peerj.7501)
Supplement: Supplemental Information 9 — Data about subsoil properties which contained 3 parallels for each treatment. The subsoil characteristics included: pH, soil organic carbon (SOC), total nitrogen (TN), the ratio of organic carbon to total nitrogen (C/N), microbial biomass carbon (MBC), microbial biomass nitrogen (MBN), the ratio of microbial biomass carbon to microbial biomass nitrogen (MBC/MBN) and ammonia nitrogen (NH4+-N). [file peerj-07-7501-s009.docx]

|  | | | | | | | | |
| --- | --- | --- | --- | --- | --- | --- | --- | --- |
| Treatments | pH | SOC | TN | CN | MBC | MBN | MBCMBN | NH_4_^+^N |
|  |  | g kg^-1^ | |  | mg g^-1^ | |  | mg g^-1^ |
| June |  |  |  |  |  |  |  |  |
| NS | 8.71 | 3.34 | 0.56 | 5.92 | 267.4 | 22.01 | 12.15 | 2.78 |
| NS | 8.83 | 2.35 | 0.63 | 3.72 | 245.05 | 24.43 | 10.03 | 2.66 |
| NS | 8.82 | 2.82 | 0.51 | 5.51 | 248.01 | 17.76 | 13.97 | 3.85 |
| NM | 8.67 | 2.86 | 0.66 | 4.34 | 173.54 | 23.26 | 7.46 | 2.6 |
| NM | 8.81 | 2.93 | 0.54 | 5.45 | 179.18 | 20.19 | 8.87 | 2.45 |
| NM | 8.79 | 3.02 | 0.77 | 3.94 | 255.43 | 24.43 | 10.45 | 2.46 |
| MS | 8.41 | 4.11 | 0.56 | 7.28 | 225.11 | 22.66 | 9.93 | 2.75 |
| MS | 8.98 | 2.91 | 0.48 | 6.02 | 365.08 | 32.28 | 11.31 | 1.76 |
| MS | 8.70 | 5.2 | 0.73 | 7.16 | 248.92 | 19.84 | 12.54 | 3.47 |
| MM | 8.85 | 3.96 | 0.82 | 4.83 | 234.62 | 22.81 | 10.29 | 4.55 |
| MM | 8.91 | 2.55 | 0.51 | 4.99 | 312.95 | 22.32 | 14.02 | 3.7 |
| MM | 9.00 | 2.9 | 0.78 | 3.72 | 310 | 15.54 | 19.95 | 2.6 |
| August |  |  |  |  |  |  |  |  |
| NS | 8.89 | 6.42 | 0.84 | 7.65 | 198 | 51.48 | 3.85 | 1.14 |
| NS | 8.97 | 5.05 | 0.69 | 7.3 | 198.6 | 50.31 | 3.95 | 0.73 |
| NS | 8.96 | 3.73 | 0.71 | 5.25 | 169.95 | 52.37 | 3.25 | 0.9 |
| NM | 8.81 | 6.18 | 0.82 | 7.5 | 171.82 | 75.96 | 2.26 | 0 |
| NM | 9.00 | 3.77 | 0.72 | 5.2 | 164.89 | 67.62 | 2.44 | 1.7 |
| NM | 8.99 | 4.47 | 0.7 | 6.4 | 237.6 | 69.28 | 3.43 | 0 |
| MS | 8.86 | 4.91 | 1.21 | 4.05 | 206.09 | 75.85 | 2.72 | 1.3 |
| MS | 8.96 | 5.38 | 1.1 | 4.9 | 149.23 | 57.51 | 2.59 | 1.64 |
| MS | 9.00 | 3.69 | 0.97 | 3.8 | 162.21 | 59.17 | 2.74 | 1.49 |
| MM | 8.86 | 4.86 | 0.65 | 7.5 | 222.17 | 68.53 | 3.24 | 0.11 |
| MM | 8.98 | 3.73 | 0.54 | 6.9 | 225.17 | 59.39 | 3.79 | 0.62 |
| MM | 8.94 | 4.74 | 0.61 | 7.8 | 228.17 | 62.24 | 3.67 | 0.92 |
| October |  |  |  |  |  |  |  |  |
| NS | 8.63 | 3.82 | 0.85 | 4.5 | 200.67 | 51.48 | 4 | 0.61 |
| NS | 8.66 | 3.52 | 0.73 | 4.8 | 200.6 | 50.31 | 4 | 0.28 |
| NS | 8.56 | 3.47 | 0.78 | 4.45 | 173.66 | 52.4 | 3.32 | 1.06 |
| NM | 8.62 | 4.1 | 0.78 | 5.3 | 182.8 | 76 | 2.41 | 1.15 |
| NM | 8.76 | 3.08 | 0.64 | 4.8 | 176.86 | 67.6 | 2.62 | 0.78 |
| NM | 8.48 | 2.36 | 0.6 | 3.95 | 249.39 | 69 | 3.6 | 1.07 |
| MS | 8.62 | 4.89 | 0.66 | 7.4 | 232.3 | 115.9 | 2.01 | 0.58 |
| MS | 8.81 | 3.6 | 0.6 | 5.75 | 176.87 | 77.51 | 2.28 | 0.44 |
| MS | 8.81 | 3.05 | 0.6 | 5.1 | 190.56 | 89.17 | 2.14 | 0.37 |
| MM | 8.81 | 3.13 | 0.63 | 5 | 233.78 | 68.53 | 3.41 | 0.74 |
| MM | 8.76 | 3.48 | 0.54 | 6.45 | 231.75 | 59.39 | 3.9 | 1.05 |
| MM | 8.86 | 4 | 0.6 | 6.55 | 236.27 | 62.24 | 3.8 | 1.4 |
| † SOC means soil organic carbon; TN means total nitrogen; C/N means the ratio of organic carbon to total nitrogen; MBC means microbial biomass carbon; MBN means microbial biomass nitrogen; MBC/MBN means the ratio of microbial biomass carbon to microbial biomass nitrogen; NH4+-N represents ammonia nitrogen. | | | | | | | | |
