# Supplementary material for: Improvement of subsoil physicochemical and microbial properties by short-term fallow practices
Source: PeerJ. 2019 Aug 19;7:e7501. doi: 10.7717/peerj.7501 (PMC6705386; doi:10.7717/peerj.7501)
Supplement: Supplemental Information 10 [file peerj-07-7501-s010.docx]

| Treatments | Topsoil TN | Subsoil TN |
| --- | --- | --- |
|  | g kg^-1^ |  |
| June |  |  |
| NS | 0.89 | 0.56 |
| NS | 0.89 | 0.63 |
| NS | 0.91 | 0.51 |
| NM | 0.77 | 0.66 |
| NM | 0.86 | 0.54 |
| NM | 0.94 | 0.77 |
| MS | 0.75 | 0.56 |
| MS | 0.67 | 0.48 |
| MS | 0.78 | 0.73 |
| MM | 0.91 | 0.82 |
| MM | 0.75 | 0.51 |
| MM | 1.02 | 0.78 |
| August |  |  |
| NS | 0.91 | 0.84 |
| NS | 0.85 | 0.69 |
| NS | 0.72 | 0.71 |
| NM | 0.78 | 0.82 |
| NM | 0.82 | 0.72 |
| NM | 0.95 | 0.7 |
| MS | 1.16 | 1.21 |
| MS | 1.28 | 1.1 |
| MS | 0.94 | 0.97 |
| MM | 0.87 | 0.65 |
| MM | 0.70 | 0.54 |
| MM | 0.77 | 0.61 |
| October |  |  |
| NS | 1.15 | 0.85 |
| NS | 1.15 | 0.73 |
| NS | 0.93 | 0.78 |
| NM | 0.93 | 0.78 |
| NM | 0.60 | 0.64 |
| NM | 0.60 | 0.60 |
| MS | 0.90 | 0.66 |
| MS | 0.78 | 0.60 |
| MS | 0.85 | 0.60 |
| MM | 0.87 | 0.63 |
| MM | 0.84 | 0.54 |
| MM | 0.87 | 0.60 |
